# SARS-CoV-2 N501Y variants of concern and their potential transmission by mouse

**DOI:** 10.1038/s41418-021-00846-4

**Published:** 2021-08-13

**Authors:** Hongyan Huang, Yichao Zhu, Zubiao Niu, Lulin Zhou, Qiang Sun

**Affiliations:** 1 Department of Oncology, Beijing Shijitan Hospital of Capital Medical University, Beijing, P. R. China; 2 Laboratory of Cell Engineering, Institute of Biotechnology, Research Unit of Cell Death Mechanism, Chinese Academy of Medical Science, Beijing, China; 3 School of Medicine, Nankai University, Tianjin, P. R. China

**Keywords:** Microbiology, Infectious diseases

The infection of severe acute respiratory syndrome coronavirus 2 (SARS-CoV-2) virus causes pneumonia of coronavirus diseases 2019 (COVID-19) [1]. A portion of patients with COVID-19 develop severe conditions that are closely related to aberrant immunity and local inflammation [2], clinically manifested as cytokine storm, expansion of myeloid-derived suppressor cells and lymphopenia, and the like [3, 4]. As of the end of March 2021, the pandemic of SARS-CoV-2 has resulted in more than 126.6 million of patients with COVID-19, leading to about 2.8 million deaths around the world (Fig. 1a) [5]. Over the past year, considerable efforts had been endeavored to implant public health measures to limit the spreading of SARS-Co-V2. However, before people started to cheer the staged success in the middle of January 2021, another wave of global SARS-CoV-2 spreading is surging up shortly after the fall-off of the second wave of pandemic (Fig. 1a). Similar to the second wave that came up with a highly transmissible variant carrying D614G mutation [6], the third wave of pandemic emerged with a number of different variants, among which three variants, B.1.1.7, B1.351, and P.1, displayed increasing transmissibility, and therefore were categorized by the world health organization (WHO) as variants of concern (VOC). By 29 March 2021, these three variants have been constituting about two-thirds of SARS-CoV-2 viruses circulating around the world (Fig. 1b) [5].

**Fig. 1:**
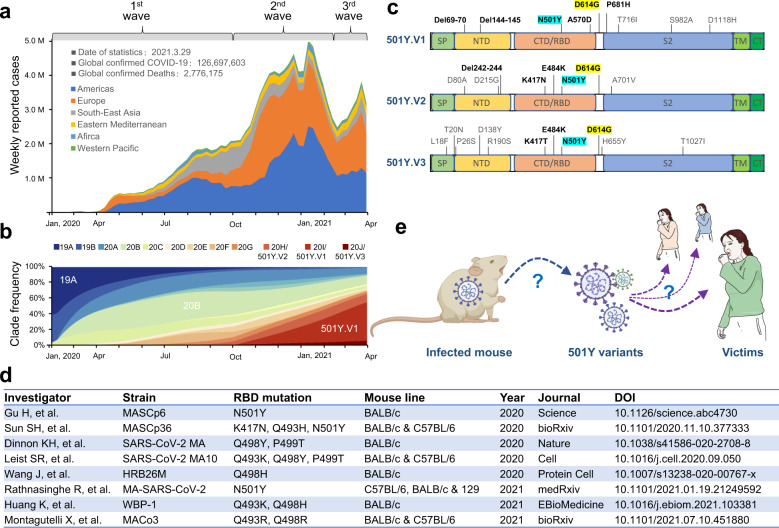
Implication of mouse-mediated transmission of SARS-CoV-2 N501Y variants in the 3rd wave pandemic. **a** Weekly cases of global confirmed COVID-19 by May 28, 2021. Data were retrieved from the COVID-19 Dashboard of the World Health Organization (WHO). **b** The proportion of different SARS-CoV-2 clades over time. The graph was made from data adopted from Nextstrain SARS-CoV-2 resources. **c** Schematic diagram depicts the mutations on spike proteins of three SARS-CoV-2 N501Y variants of concern. Key mutations are in black and bold. Shared mutations are shadowed. SP signal peptide, NTD N terminus domain, CTD/RBD C terminus domain/receptor-binding domain, TM transmembrane region, CT cytoplasmic terminus. **d** A list of SARS-CoV-2 strains, harboring mutations in the RBD region of S glycoprotein, that could effectively infect wild-type mice. **e** Schematic demonstration of the mouse as a transmitter of the SARS-CoV-2 N501Y variants of concern.

## The N501Y variants of concern

The B.1.1.7 lineage, also known as 20I/501Y.V1 or VOC 202012/01, was first detected in the United Kingdom (UK) in December 2020; it demonstrates a substantial transmission advantage over other non-VOC variants with a 50–100% higher reproduction number and has been identified in at least 130 countries [5, 7]. The B.1.351 lineage, also known as 20H/501Y.V2 or VOC 202012/02, was first identified in South Africa (SA) in December 2020; this variant spread rapidly, leading to the displacement of other lineages in multiple SA regions within weeks. The P.1 lineage, also known as 20J/501Y.V3 or B.1.1.28, was identified in December 2020 in four travelers to Japan from Brazil; this variant is a dominant circulating variant in Brazil (>72%), and has been detected in at least 45 countries [5, 7]. The high transmissibility of these variants was attributed, at least partially, to the acquired mutations in the receptor-binding domain (RBD) of spike (S) protein (Fig. 1c), which is responsible for the binding to the angiotensin-converting enzyme-2 (ACE2) receptor.

## Impacts on detection, immunity, and infectivity

The mutations in S protein could have profound impacts on diagnostic detection, antiviral immunity, and virus infectivity, all of which could actually contribute to the rapid spreading of above VOCs. It was shown that the deletion at positions 69 and 70 of the S glycoprotein (ΔH69-V70) of 501Y.V1 variant was associated with diagnostic test failure for the ThermoFisher TaqPath probe targeting the spike protein, a phenomenon termed S-gene target failure (SGTF) [8]. Meanwhile, the other two VOCs, 501Y.V2 and 501Y.V3, demonstrated immune escape from the neutralization of either convalescent and vaccine sera, or neutralizing antibodies; the E484K and N501Y mutations in RBD seemed to primarily contribute to the immune escape [9]. Currently, the influences of the mutations on variants’ infectivity are an open question in debate. Some investigations strongly supported an enhanced binding of mutant RBD to human ACE2 protein, favoring an idea of increasing infectivity of these VOCs to human cells. However, some others showed that viruses pseudotyped with the VOCs’ S glycoprotein displayed reduced infectivity in cells expressing human ACE2 [9, 10], suggesting that alternative mechanisms may facilitate the rapid spreading of 501Y VOCs.

## Mouse infection by N501Y SARS-CoV-2

In addition to human–human transmission, animals had been implicated in the transmission of SARS-CoV-2 viruses. For example, an investigation on 16 mink farms in the Netherlands indicated that 68% of tested individuals had evidence for SARS-CoV-2 infection, some of them turned out to be infected with strains from minks. Moreover, cat, dog, ferret, hamster, tiger, and lion were also capable of being infected by the early SARS-CoV-2 strain [11]. Interestingly, by screening a panel of cells expressing ACE2 of 14 species with viruses pseudotyped with different S mutants, Li et al. found that the S proteins of three 501Y VOCs endowed increased infectivity selectively in cells expressing mouse ACE2, which was likely associated with the mutations of N501Y, E484K, and K417N in the RBD region [9]. In agreement with Li’s results, we found that N501Y mutation alone could render effective binding of the 501Y-RBD to mouse ACE2, leading to fusion of neighboring membranes and effective infection; while the wild-type S glycoprotein carrying N501 could not bind to mouse ACE2 to mediate membrane fusion and virus infection at all [10]. These results suggest that the N501Y mutation may break the cross-species barrier to establish effective infection in mouse. Consistently, Rathnasinghe et al. recently obtained a mouse-adapted SARS-CoV-2 strain (MA-SARS-CoV-2) by serial passaging a clinical virus isolate (USA-WA1/2020) in mice; the MA-SARS-CoV-2 coincidently harbors a N501Y mutation in addition to two other S mutations (Fig. 1d) [12]. Moreover, Gu et al. independently isolated another mouse-adapted strain (MASCp6) that has N501Y as the only mutation in the S glycoprotein, the MASCp6 was able to effectively infect mouse lung (Fig. 1d) [13]. Though mutations other than N501Y were also identified in mouse-adapted or genetically engineered SARS-CoV-2 strains (Fig. 1d), they were not reported in the circulating human variants except for the K417N mutation that, coming up with the further passaging of MASCp6, is present in 501Y.V2 and 501Y.V3 (Fig. 1d), their potential implications in SARS-CoV-2 spreading in human warrant further investigation. Together, it is conceivable that mice are likely susceptible to the newly emerging N501Y VOCs.

## Implications of mice as a potential transmitter

Mice are known hosts of a number of infectious diseases, such as pestis, epidemic hemorrhagic fever, bacillary dysentery, and salmonellosis. Considering that mice are widely existing and hard to be controlled, particularly in communities of poor sanitation, and may cause catastrophic impacts if being a N501Y VOCs transmitter (Fig. 1e), further investigations are warranted to clarify the role of mice in SARS-CoV-2 transmission. First, authentic SARS-CoV-2 virus of 501Y VOCs should be tested to examine whether mice could be effectively infected, if possible, other emerging variants should be tested too; second, co-housing infected mice with naive mice, and primates such as monkey or chimpanzee as well, to examine the feasibility of mouse-to-mouse and mouse-to-human transmission, meanwhile, clarify the possible routes whereby viruses were transmitted if transmittable (Fig. 1e); third, epidemiological studies should be implanted in regions with COVID-19 patients, such as the outbreak communities and hospitals, to examine whether mice are infected with SARS-CoV-2, genome sequencing would be necessary to confirm virus identity. In case sufficient evidences supporting mice are bona fide transmitters of 501Y VOCs, stricter measures, in addition to the existing public preventive measures [14], should be taken to limit mouse-mediated transmission. This will include, but not limited to, (1) extinguishing the mouse with routine measures such as mouse poison and trap; (2) improving environmental sanitation with emphasis on areas such as dining rooms, garage stations, transport stations, airports and sewage system; (3) enhancing environmental surveillance for the presence of SARS-CoV-2 virus; (4) monitoring the emergence of novel variants evolved from the mouse that are highly contagious to human.
